# Implementation of low-dose buprenorphine induction at a syringe services program

**DOI:** 10.1186/s13722-026-00661-7

**Published:** 2026-04-06

**Authors:** Maia H. Hauschild, Peyton V. Warp, William H. Eger, Ryan Hood, Monica Bahamon, David W. Forrest, Tyler S. Bartholomew, Edward Suarez, Teresa A. Chueng, Katrina J. Ciraldo, Hansel E. Tookes, David P. Serota

**Affiliations:** 1 Division of Infectious Diseases, Department of Medicine, University of Miami Miller School of Medicine, Miami, USA; 2 Division of Infectious Diseases & Global Public Health, School of Medicine, University of California San Diego, La Jolla, CA USA; 3 School of Social Work, San Diego State University, San Diego, CA USA; 4 Department of Public Health Sciences, University of Miami Miller School of Medicine, Miami, USA; 5 Department of Psychiatry and Behavioral Sciences, University of Miami Miller School of Medicine, Miami, USA; 6 Department of Family Medicine and Community Health, University of Miami Miller School of Medicine, Miami, USA; 7 Department of Obstetrics, Gynecology and Reproductive Sciences, University of Miami Miller School of Medicine, Miami, USA

**Keywords:** Addiction, Opioid use disorder, Fentanyl, Buprenorphine, Low-dose induction, Microdose, Syringe services program

## Abstract

**Background:**

Fentanyl’s penetration into the unregulated drug supply has complicated the treatment of opioid use disorder (OUD), particularly by increasing the risk of buprenorphine-precipitated opioid withdrawal (BPOW). Buprenorphine, a partial opioid agonist, remains a first-line treatment for OUD, but traditional induction methods can be intolerable for people using fentanyl. Low-dose induction (LDI), a strategy characterized by gradual buprenorphine titration without prior withdrawal, has emerged as a promising alternative to mitigate BPOW. However, the feasibility and acceptability of LDI in low-barrier, real-world settings such as syringe services programs (SSPs) remain underexplored.

**Methods:**

We conducted a mixed-methods prospective cohort study from June 2023–2024 at an SSP in Miami, Florida, offering a 4-day LDI protocol to patients with OUD who were interested in starting buprenorphine. Follow-up, conducted on a walk-in basis within four weeks, included urine drug screens (UDS), symptom surveys and semi-structured qualitative interviews. The primary outcome was successful buprenorphine initiation, defined by a positive UDS for buprenorphine at follow-up.

**Results:**

Of the 30 participants enrolled in the 4-day LDI protocol, most (*n* = 29) had prior buprenorphine experience and nearly 90% (*n* = 26) reported past BPOW. Only 16 (53%) returned for follow-up. Nine (56%) of those followed up tested positive for buprenorphine, 11 (68.8%) reported that LDI worked for them, and 12 (75%) said they would use the method again. Qualitative interviews revealed six key themes: (1) LDI mitigates withdrawal symptoms; (2) instructions were helpful but could be improved; (3) fear of BPOW motivated LDI use; (4) LDI enabled autonomy in recovery; (5) unstable living environments hindered adherence; and (6) LDI allowed participants to maintain social roles.

**Conclusions:**

While only 30% of the cohort had objective evidence of buprenorphine induction, most reported successful attempts and found LDI acceptable and empowering. High loss to follow-up and environmental instability limited our conclusions in this outpatient harm reduction setting. Further research is needed to refine LDI protocols and address the structural determinants affecting treatment success among people who use fentanyl.

## Background

Illicitly manufactured fentanyl exacerbated the national overdose crisis when it replaced heroin as the dominant opioid in several major illicit drug markets in North America in the second decade of the 21st century [1]. While national drug overdose rates began to decline in 2023 for the first time since 2018, there were still greater than 48,000 overdose deaths related to fentanyl in 2024 [2]. Safe and effective medications for opioid use disorder (MOUD), including the partial opioid agonist buprenorphine, have resulted in reductions in overdose and serious opioid-related acute care use [3]. Beyond systemic barriers to treatment, people with opioid use disorder (OUD) face the risk of buprenorphine-precipitated opioid withdrawal (BPOW). BPOW is a phenomenon in which opioid withdrawal symptoms are paradoxically worsened by the initiation of buprenorphine due to its activity as a high-affinity partial-agonist at the mu-opioid receptor. Reported rates of BPOW in the literature vary substantially, between 1 and 22% [4, 5]. The lipophilicity of fentanyl, which prolongs tissue retention, has been proposed as a contributor to BPOW and necessitates the urgent need for new ways to initiate buprenorphine in the fentanyl era [6]. Multilevel challenges to starting buprenorphine contribute to low treatment initiation and retention as well as high mortality among people with OUD, especially in the context of high fentanyl adulteration in unregulated opioid supplies [7].

The American Society of Addiction Medicine’s clinical practice guidelines favor waiting until patients experience objective signs of opioid withdrawal before taking the first dose of buprenorphine to avoid BPOW [8]. Under this model, nausea, vomiting, diarrhea, anxiety and pain are expected. An alternative approach, often referred to as microdose-induction or low-dose induction (LDI), has emerged to remove the withdrawal prerequisite for buprenorphine initiation. LDI involves administering small escalating doses of buprenorphine with overlapping full opioid agonists to prevent severe withdrawal symptoms and reduce the risk of BPOW [9]. The efficacy of LDI, particularly for individuals averse to withdrawal or using fentanyl, has been demonstrated in inpatient settings with close clinical supervision [10].

While case reports have suggested the feasibility and success of LDI in outpatient substance use disorder clinics [11], a larger study by *Suen et al.* found only 34% of people using an LDI protocol successfully initiated buprenorphine in the outpatient setting [12]. While various LDI protocols are well-documented [11, 13, 14], there remains a critical need to determine best practices for LDI implementation in low-barrier harm reduction settings, like syringe services programs (SSPs), to increase buprenorphine uptake and retention for the most vulnerable patients, including those experiencing homelessness.

To address this gap, we adapted a 4-day LDI treatment protocol and began offering patients the LDI buprenorphine initiation at an SSP in Miami, Florida [15, 16]. In this mixed-methods study, we aimed to evaluate the preliminary clinical outcomes of LDI for initiating buprenorphine while examining implementation outcomes (e.g., feasibility, acceptability) in a real-world harm reduction setting [17].

## Methods

### Study design and setting

We conducted a prospective cohort study from June 2023 to June 2024 of patients with a diagnosis of moderate to severe OUD per the *Diagnostic and Statistical Manual of Mental Disorders* (Fifth Edition) seeking buprenorphine from their local SSP (IDEA Miami). The SSP provides harm reduction services, wound care, and substance use disorder treatment services, including buprenorphine for OUD, to individuals regardless of insurance status [15]. The Institutional Review Board at the University of Miami approved this study (UM IRB #20230483) and written informed consent was obtained from participants. Strengthening the Reporting of Observational Studies in Epidemiology (STROBE) reporting guidelines were followed.

### Recruitment and data collection

**Fig. 1:**
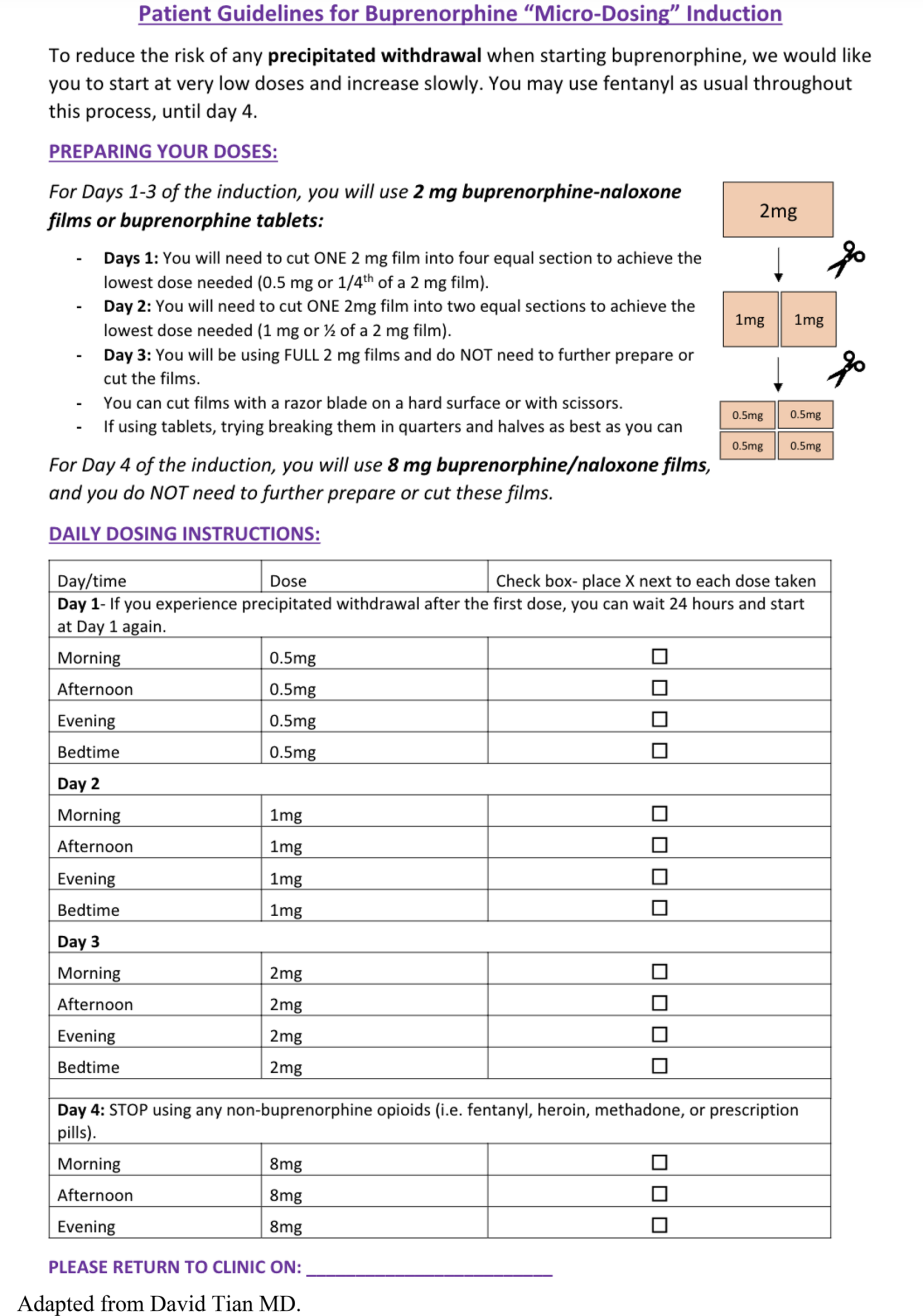
LDI handout

.Eligible participants were adults aged 18 years or older with moderate to severe OUD who tested negative for buprenorphine by urine drug screen (UDS) while presenting for buprenorphine induction at the SSP’s weekly student-run free clinic. The SSP provides OUD treatment five days per week including via telehealth, but study screening was limited to individuals presenting to the student-run clinic session. Once screened, participants were recruited by trained medical student staff members and physicians. Once screened, participants were recruited by trained medical student staff members and physicians. Individuals interested in participating provided written informed consent and met with a clinician to review their substance use history and goals for treatment. Participants were presented with options for buprenorphine initiation, including LDI and traditional initiation, as well as referral to methadone treatment. For participants who completed the informed consent process, physicians provided counseling on the 4-day LDI protocol and an instructional handout (Fig. 1) [18, 19]. We chose a short LDI protocol because preliminary evidence suggests no difference in LDI success between 4 and 7-day protocols [12]. Because of the housing instability and high intensity drug use of our patient population, we felt that use of the shortest possible protocol would be advantageous. Prescriptions for buprenorphine (a 30-day supply) and medications to alleviate any withdrawal symptoms were sent to an affiliated outpatient pharmacy. Results of a 13-panel UDS, in addition to xylazine testing results were also recorded.

Besides the UDS results, all data collected were self-reported; at the conclusion of the initial clinical visit, participants completed an interviewer-administered baseline survey assessing demographic characteristics and prior experiences with buprenorphine. In the survey, participants rated withdrawal symptoms associated with past buprenorphine induction experiences on a scale of mild to severe. Participants were compensated $10 for completion of the initial study visit. Participants were asked to return to the clinic within one week, but up to four weeks both for clinical follow up and for follow-up study procedures.

At the follow-up visit, a UDS, a survey about participants’ withdrawal symptoms and semi-structured qualitative interviews were completed for each participant. The research team developed semi-structured interview guides aimed to identify opportunities to support SSP clients in starting buprenorphine. Primary domains included (1) experiences with LDI protocol (2) comparison to past experiences starting buprenorphine. All interviews took place in person in a confidential space and were conducted by trained student researchers. Interviews were audio-recorded, professionally transcribed and reviewed by the research team for accuracy. Participants received an additional $10 for completing all follow-up procedures.

### Outcomes of interest

The primary outcome was successful buprenorphine initiation, defined as having a UDS positive for buprenorphine at the follow-up visit. Treatment success is presented for those who attended a follow-up visit as well as for the whole cohort, where loss to follow-up was considered unsuccessful treatment.

### Data analyses

Descriptive statistics are presented as percentages for categorical variables and medians and interquartile ranges for continuous variables. Analyses were completed using Microsoft Excel (Version 16.96).

Our qualitative analysis process for follow-up interviews followed an “open-coding” process informed by the Capability, Opportunity, Motivation, and Behavior (COM-B) framework. Structuring thematic analysis around the COM-B components allowed researchers to systematically identify barriers and facilitators to behavior change as reported by participants, allowing inductive themes to emerge from the data. COM-B was chosen for its structured approach to developing targeted interventions, which involves evaluating individuals’ capability, motivation, and opportunity to engage in a specific behavior, while accounting for the broader contextual factors that influence change [20]. Three graduate-level qualitative analysts (MHH, PVW, WHE) first read small batches of transcripts to gain a broad understanding of topics covered and to develop a series of memos to clarify emergent ideas and dominant COM-B constructs. Qualitative memos were developed for approximately one-third of transcripts to ensure no additional inductive ideas were present and to develop a preliminary codebook. After development of the preliminary codebook, which included specific inclusion and exclusion criteria, two team members met biweekly to address discrepancies in their independent coding process and to further refine the codebook until coding consensus —defined as consistent application of the codes to transcripts—was established. Coding consensus was achieved after approximately 20% of transcripts were double-coded and reviewed through consensus discussions. A third analyst was consulted in the rare instances where consensus could not be reached between the initial coders. Once all transcript data was coded, the coded passages were read by all three qualitative analysts to identify themes. The team refined the final themes through discussions with the rest of the research team, which are presented below. All coding was completed using NVivo 14 (QSR International Pty Ltd, 2020).

## Results

### Baseline characteristics

From June 2023 to June 2024, 45 participants presenting to the SSP to start buprenorphine were approached for enrollment in the study. Thirty participants seeking LDI buprenorphine initiation were enrolled (median age = 35, 53% male). Nearly half of participants self-reported being unhoused (47%) and a hepatitis C diagnosis (47%), and 37% reported a post-traumatic stress disorder diagnosis (Table 1). UDS results at baseline were 97% positive for fentanyl, 76% for cocaine, and 64% for xylazine.

**Table 1:** Participant demographics and health characteristics at baseline and follow-up

Variable	Baseline (*N* = 30)	Follow-Up (*N* = 16)
	*N* (%)	*N* (%)
**Age (median**,** Q1–Q4)**	35 (32–40)	35 (30.5–39)
**Gender**		
Man	16 (53.3%)	11 (68.8%)
Woman	14 (46.7%)	5 (31.3%)
**Race/Ethnicity**		
White non-Hispanic	16 (53%)	8 (50%)
White Hispanic	12 (40%)	7 (43.8%)
Black Hispanic	1 (3%)	1 (6.3%)
Native American	1 (3%)	0 (0%)
**Housing Status**		
Apartment/House	16 (53.3%)	8 (50%)
Unhoused	14 (46.7%)	8 (50%)
Street/camping/squatting	9 (64.3%)	5 (31.3%)
Couch surfing	5 (35.7%)	3 (18.8%)
**Educational Attainment**		
Graduated high school	24 (80%)	12 (75%)
Did not graduate high school	6 (20%)	4 (25%)
**Smokes Tobacco**		
Yes	25 (83.3%)	13 (81.3%)
No	5 (16.7%)	3 (18.8%)
Employment Status*		
Unemployed	20 (66.7%)	11 (68.8%)
Employed	7 (23.3%)	3 (18.8%)
Unable to work	2 (6.7%)	1 (6.3%)
**Lifetime Diagnoses (self-report)**		
Hepatitis C	14 (46.7%)	8 (50%)
PTSD	11 (36.7%)	5 (31.3%)
Generalized anxiety disorder	10 (33.3%)	4 (25%)
Major depressive disorder	10 (33.3%)	4 (25%)
ADHD	8 (26.7%)	3 (18.8%)
Asthma	6 (20%)	4 (25%)
Bipolar disorder	5 (16.7%)	2 (12.5%)
Chronic wounds/ulcers	5 (16.7%)	3 (18.8%)
HIV	0 (0%)	0 (0%)
**UDS Results at Baseline**		
Fentanyl	29 (96.7%)	13 (81.3%)
Cocaine	22 (73.3%)	12 (75%)
**Buprenorphine**	**0 (0%)**	**9 (56%)**
Xylazine**	18 (64.3%)	4 (25%)
MDMA	11 (36.7%)	7 (43.8%)
Cannabis	10 (33.3%)	7 (43.8%)
Benzodiazepines	8 (26.7%)	6 (37.5%)
Amphetamine	5 (16.7%)	2 (12.5%)
Opiates	5 (16.7%)	7 (43.8%)
Ethyl glucuronide	4 (13.3%)	2 (12.5%)
Methamphetamines	4 (13.3%)	2 (12.5%)
Methadone	2 (6.7%)	0 (0%)
Oxycodone	0 (0%)	1 (6.3%)
Barbiturates	0 (0%)	0 (0%)

*One participant did not respond to this question in the baseline survey

**28 participants were tested for xylazine

Note: PTSD, post-traumatic stress disorder; ADHD, attention deficit hyperactivity disorder; HIV, human immunodeficiency virus; MDMA, 3,4-methylenedioxymethamphetamine

Most participants (80%) reported previous buprenorphine treatment experience and fewer than 5 lifetime induction attempts (79%) (Table 2). 90% (90%) reported ever experiencing BPOW in the past. The most reported severe symptoms of opioid withdrawal were anxiety (83%), bone/joint pain (73%), sweating (70%) and insomnia (70%). Over half reported experiencing severe nausea (63%), runny nose (63%), mood change (60%), vomiting (53%), and shakes/tremor (53%) (Table 2). Before study enrollment, fewer than half (43%) of participants were familiar with LDI and 20% had previously attempted to use an LDI method (Table 2).

**Table 2:** Experiences with buprenorphine

Variable	At Baseline (*N* = 30)	After LDI (*N* = 16)	
	*N* (%)	*N* (%)	
**Previous buprenorphine induction attempts**			
≤ 5 attempts	23 (79.3%)		
> 5 attempts	6 (20.7%)		
**Previous buprenorphine treatment duration**	24 (80%)		
Duration*			
≤ 1 year	14 (60.9%)		
> 1 year	9 (39.1%)		
**Experienced precipitated withdrawal**			
Yes	26 (89.6%)	7 (44%)	
No	3 (10.3%)	9 (56%)	
**Withdrawal symptoms described as severe**			
Anxiety	25 (83.3%)	8 (50%)	
Bone/joint pain	22 (73.3%)	5 (31.3%)	
Insomnia	21 (70%)	6 (37.5%)	
Sweating	21 (70%)	5 (31.3%)	
Mood change	18 (60%)	5 (31.3%)	
Runny nose	19 (63.3%)	4 (25%)	
Nausea	19 (63.3%)	3 (18.8%)	
Shakes/tremors	16 (53.3%)	3 (18.8%)	
Vomiting	16 (53.3%)	1 (6.3%)	
Loss of appetite	12 (40%)	5 (31.3%)	
Diarrhea	14 (46.7%)	1 (6.3%)	
**Heard of LDI/ “microdosing” before**	13 (43.3%)		
Source of information (*n* = 13)			
Friend	7 (23.3%)		
Doctor/clinic	5 (16.7%)		
Internet	1 (3.3%)		
**Tried LDI before**	6 (20%)		
**Agreement with the following statements**:			
***Baseline Survey***			
Confident in ability to get onto buprenorphine using LDI	28 (93.3%)		
Confident in ability to stay on buprenorphine after LDI	28 (93.3%)		
Heard that microdosing eliminates precipitated withdrawal	13 (43.3%)		
Knows someone who has used LDI to get onto buprenorphine bupbuprenorphine	10 (33.3%)		
Is skeptical about LDI	10 (33.3%)		
Knows people who have tried LDI	12 (40%)		
Wanted to try LDI but didn’t know how	9 (30%)		
Knows someone who tried LDI but was unsuccessful	4 (13.3%)		
***Follow-up survey***			
The LDI method worked for me		11 (68.8%)	
I reached my goal dose of buprenorphine		6 (37.5%)	
Median days to initiation (Q1–Q4)		4 (4–6.25)	
Instructions were easy to follow		13 (81.3%)	
Fewer withdrawal symptoms than past attempts		11 (68.8%)	
Would use LDI again		12 (75%)	
Would recommend LDI to a friend		12 (75%)	
Felt motivated to continue buprenorphine		13 (81.3%)	
Followed LDI instructions		10 (62.5%)	
LDI was more comfortable than past attempts		14 (87.5%)	

*One participant did not respond to this question in the baseline survey

Note: LDI, low-dose induction

### Quantitative outcomes

Sixteen (52%) participants completed a follow-up assessment after a median of 17.5 days from baseline (IQR 7–35 days) and fifteen (50%) completed follow-up interview. Of the 16 who completed follow-up, nine (56%) tested positive for buprenorphine (30% of the total cohort) and 11 (69%) endorsed that they found the protocol to be effective; however, only 6 (38%) of participants attested to reaching their goal dose of buprenorphine (Table 3). The most reported severe withdrawal symptom was anxiety (50%), followed by insomnia (38%). Most (75%) of participants stated they would use LDI again as a future buprenorphine initiation strategy.

**Table 3:** Qualitative themes according to COM-B analysis

COM-B Domain	Theme
Physical capability	LDI attenuates physical and psychological opioid withdrawal symptoms
Psychological capability	LDI instructions were generally helpful, informative, and simple; however, some participants requested additions
Automatic motivation	Fear and anxiety surrounding BPOW motivates an LDI attempt
Reflective motivation	Outpatient LDI empowers individuals to engage in deliberate decision-making and set their own recovery goals
Physical opportunity	Lack of stable environment and structured support undermines successful LDI in street-based contexts.
Social opportunity	LDI allows individuals to maintain a regular schedule and social role compared to standard induction protocols.

Note: COM-B, capability, opportunity, motivation and behavior; BPOW, buprenorphine precipitated withdrawal; LDI, low-dose induction

### Qualitative themes

#### Physical capability

***Theme 1: LDI attenuates physical and psychological opioid withdrawal symptoms***

When compared to past attempts at buprenorphine induction, participants described a faster, easier, and *“definitely more comfortable”* experience starting buprenorphine with LDI. In traditional inductions, participants described an *“excruciating”* first *“48 to 72 hours of cold turkey”* in which they were *“full-blown sick*,* throwing up and all that.”* Another participant stated of traditional induction, “*I had lost a lot of weight and couldn’t eat for a week.”* Comparatively, with LDI, most participants reported mild, if any, withdrawal symptoms, as supported by one participant who mentioned he *“wasn’t getting any withdrawal symptoms*.”

#### Psychological capability

***Theme 2: LDI instructions were generally helpful***,*** informative***,*** and simple; however***,*** some participants requested additions***

Beyond being helpful, some participants described the instructions as essential, noting “*without the paper*,* I probably would’ve been a little lost.”* Participants noted that the instructions were not enough to understand LDI alone and state that verbal explanations were essential. One participant stated, “*since you guys thoroughly explained everything to me*,* I was able to do this.”* Some participants requested additions to the instructions, such explicit counseling on concurrent drug use. For example, one participant recommended including instructions to *“cut the fentanyl less and less”* as the days progress; another requested instruction on *“when to take the buprenorphine and when to take the opiate.*”

#### Automatic motivation

***Theme 3: Fear and anxiety surrounding BPOW motivates an LDI attempt***

Even participants who never experienced precipitated withdrawal in their lifetime described fear and anxiety surrounding the phenomenon; one stated *“I’ve been through the withdrawals so many times now that I know [they] are really scary and will really*,* really hurt you.”* Another participant stated:

*Just the fact that it’s microdosing*,* and you can do both at the same time*,* it takes away*.

*the fear of that withdrawal effect*,* because the precipitated withdrawals are horrible.*

*Which*,* I can’t even really say that I’ve ever actually experienced it*,* but [it’s] the fear that they put in you*

#### Reflective motivation

***Theme 4: Outpatient LDI empowers individuals to engage in deliberate decision-making and set their own recovery goals***

Many participants felt ready to quit fentanyl, but not other substances, and appreciated the flexibility of outpatient LDI versus an inpatient medically supervised detox program. One participant stated, *“I can worry about quitting pot another time [because] it’s not heroin and it’s not fentanyl.”* Despite verbal counseling to adhere to the dosing regimen on the LDI handout, participants appreciated their autonomy in the ability to tailor buprenorphine dosing to their withdrawal symptoms. Notably, participants took pride in the ability to plan their dosing regimen; one described LDI as “*more empowering*” than past induction experiences while another concluded his interview with the proud statement “*I just did it—like Sinatra said*,* “I did it my way.*”

#### Physical opportunity

***Theme 5: Environmental instability and structured support undermined MOUD induction***,*** including LDI***,*** for people experiencing homelessness***

Participants experiencing homelessness noted the difficulty of adhering to the LDI protocol, stating *“you’re not getting high from [buprenorphine] and you just wanna block out the pain and the anxiety and the stress from being on the streets.”* One reflected on how difficult it was to start buprenorphine surrounded by other people who were *“shooting up*” on the streets, stating that *“a big thing is just the places and the people.”* In addition, participants reported self-directed use of street drugs or other substances to alleviate withdrawal symptoms. One participant stated that cannabis “*really help[ed] the cravings*” for fentanyl. Others reported using street fentanyl to manage pain. Another returned to alcohol use after a year of abstinence to manage pain during the withdrawal period. While these lived experiences do not specifically speak to the difficulties of LDI for all people experiencing homelessness, they speak to the difficulty of MOUD induction when self-treating mental and physical pain in a challenging context.

#### Social opportunity

***Theme 6: LDI allows individuals to maintain a regular schedule and social role compared to standard induction protocols***

Avoiding withdrawal by continuing full agonist opioid use during the LDI appeared to decrease interruptions in daily life for people who experience florid withdrawal prior to initiating buprenorphine. As one participant stated that he was “*able to concentrate*” throughout the LDI protocol, which enabled him to “*actually provide… help for those who [were] depending on [him]*.” The ability to go to work allowed some participants to receive social support through the workplace during LDI; one participant stated that his boss encouraged him: *“you’re fighting the good fight*,* man. I see your arm’s looking better. You’re healin’ up.”*

## Discussion

This study examined the implementation of a 4-day LDI protocol at a low barrier, SSP-based buprenorphine program for PWID using predominantly fentanyl. While acceptability of the LDI protocol was high, feasibility was limited, as nearly half of all participants were lost to follow-up and only one-third of participants had objective evidence of recent buprenorphine use. These data reflect challenges in managing MOUD among PWID with high rates of homelessness and co-occurring stimulant and xylazine use. While prior studies of LDI in the inpatient setting and case series in the outpatient setting have demonstrated high success rates [10, 12], our data highlight significant barriers to success of LDI in real-world harm reduction settings.

**Fig. 2:**
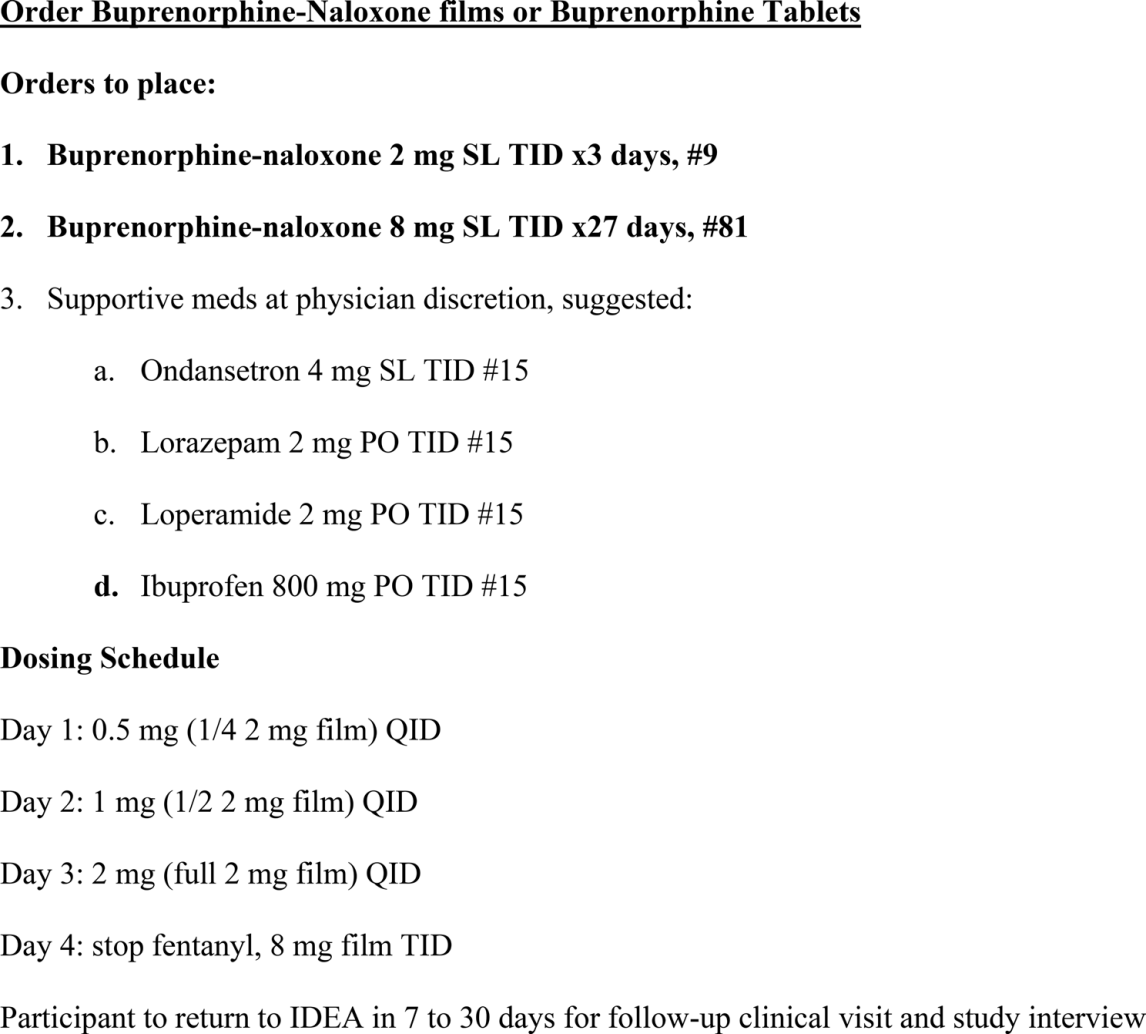
Physician orders

There was a discrepancy between subjective and objective success with the LDI protocol. When looking at our primary outcome of buprenorphine on follow-up UDS, one may interpret LDI at this low-barrier buprenorphine program with mixed success—although fentanyl can remain positive for > 28 days after last use, more participants (81%) tested positive for fentanyl at follow-up than buprenorphine (53%) (Table 1). The first study of outpatient LDI attempt similarly found low initiation (34%) and retention (21%) rates [12]. Even with induction options, extensive counseling, and take-home supportive medications for withdrawal symptoms (Fig. 2), most participants did not stop using fentanyl. However, in fealty to harm reduction principles, any positive change (e.g., presence of buprenorphine or reduced use) should be viewed as a success and celebrated.

One difference between this study and that of *Suen et al.*. is the definitions of successful buprenorphine initiation; Suen defined success as “self-reported LDI completion.” [12] Our study adds objective findings of buprenorphine in UDS and adds rich qualitative evidence to explore the personal motivations, social determinants, and support networks that determine successful buprenorphine induction in a harm reduction setting. In our self-reported measures of LDI success, we found that over two-thirds of participants agreed that LDI worked for them and would use the method again. Somatic and psychological withdrawal symptoms were numerically lower with LDI compared to previous attempts (Tables 2 and 3); however, severe psychiatric withdrawal symptoms, including insomnia and anxiety, were still experienced by over half of participants. Understanding the nuances between objective and subjective study outcomes will warrant further qualitative studies to understand barriers to LDI in a harm reduction setting, especially for people with low resources.

Qualitative themes further supported higher rates of self-reported successful buprenorphine initiation in our cohort. Participants were motivated to try LDI because they were fearful of the suffering from withdrawal required by traditional induction methods, and overall they found the experience to be more tolerable compared to past experiences. Differences in success rates could be further attributed to a diversity of LDI protocols used in different settings.

In considering the gap in effectiveness between inpatient LDI and outpatient LDI, we suspect social determinants of health as the primary drivers of unsuccessful treatment. On a national level, policies preventing prescription of full opioid agonists (e.g., methadone) in the outpatient setting for OUD caused participants to rely on an unstable and unregulated drug supply during LDI. Housing instability and local residential addiction treatment program policies which prohibit buprenorphine [21], undermine PWID’s ability to safely store or access their medication. Lacking proper shelter, nutrition, and support is undoubtedly a barrier to long-term adherence to buprenorphine treatment.

The results of this study must be interpreted in the context of several limitations. The high loss to follow-up biased the results toward individuals who successfully completed LDI. While we interpreted the primary outcome conservatively, where loss to follow-up was considered as being negative for buprenorphine, the qualitative results are not representative of the cohort overall due to low retention at the follow-up visit. Recall bias and social desirability bias could have influenced validity of qualitative findings. Beyond attrition and recall bias, this study is limited by failure to include individuals living with HIV and lack of diversity with respect to participant race and ethnicity; the majority were non-Hispanic White. This study is further limited by a small sample size and lack of a contemporary control group to evaluate how the LDI protocol compares directly to a traditional induction strategy, although data from our SSP indicate a 59% 3-month retention rate on buprenorphine [14]. A higher 3-month retention rate compared to this study may be attributed to measuring buprenorphine prescriptions picked up at the pharmacy as opposed to UDS results.

## Conclusion

This study addresses a critical gap in qualitative evidence of LDI implementation within harm reduction settings. LDI provides an alternative, humane pathway to starting buprenorphine which participants not only accept but find empowering. Fear of precipitated withdrawal motivated participants to attempt LDI, and they reflected positively on their autonomy in creating their own dosing and recovery goals while maintaining social responsibilities. This mixed-methods study demonstrates higher self-reported success with LDI compared to prior studies in non-harm reduction settings, underscoring the need for additional implementation research to optimize delivery of LDI in this low barrier context.

## Data Availability

The datasets generated and analyzed during the current study are available from the corresponding author on reasonable request.

## Abbreviations

OUD: Opioid use disorder
MOUD: Medications for opioid use disorder
BPOW: Buprenorphine precipitated opioid withdrawal
LDI: Low-dose induction
PWID: People who inject drugs
SSP: Syringe services program
UDS: Urine drug screen
COM-B: Capability, opportunity, motivation, and behavior
